# Implication of TLR- but Not of NOD2-Signaling Pathways in Dendritic Cell Activation by Group B *Streptococcus* Serotypes III and V

**DOI:** 10.1371/journal.pone.0113940

**Published:** 2014-12-01

**Authors:** Paul Lemire, David Roy, Nahuel Fittipaldi, Masatoshi Okura, Daisuke Takamatsu, Eugenia Bergman, Mariela Segura

**Affiliations:** 1 Laboratory of Immunology, Faculty of Veterinary Medicine, University of Montreal, St-Hyacinthe, Quebec, Canada; 2 Public Health Ontario, Toronto, Ontario, Canada; 3 Department of Laboratory Medicine and Pathobiology, Faculty of Medicine, University of Toronto, Toronto, Ontario, Canada; 4 Bacterial and Parasitic Diseases Research Division, National Institute of Animal Health, National Agriculture and Food Research Organization, Tsukuba, Ibaraki, Japan; 5 The United Graduate School of Veterinary Sciences, Gifu University, Gifu, Japan; Columbia University, United States of America

## Abstract

Group B *Streptococcus* (GBS) is an important agent of life-threatening invasive infection. It has been previously shown that encapsulated type III GBS is easily internalized by dendritic cells (DCs), and that this internalization had an impact on cytokine production. The receptors underlying these processes are poorly characterized. Knowledge on the mechanisms used by type V GBS to activate DCs is minimal. In this work, we investigated the role of Toll-like receptor (TLR)/MyD88 signaling pathway, the particular involvement of TLR2, and that of the intracellular sensing receptor NOD2 in the activation of DCs by types III and V GBS. The role of capsular polysaccharide (CPS, one of the most important GBS virulence factors) in bacterial-DC interactions was evaluated using non-encapsulated mutants. Despite differences in the role of CPS between types III and V GBS in bacterial internalization and intracellular survival, no major differences were observed in their capacity to modulate release of cytokines by DC. For both serotypes, CPS had a minor role in this response. Production of cytokines by DCs was shown to strongly rely on MyD88-dependent signaling pathways, suggesting that DCs recognize GBS and become activated mostly through TLR signaling. Yet, GBS-infected TLR2^-/-^ DCs only showed a partial reduction in the production of IL-6 and CXCL1 compared to control DCs. Surprisingly, CXCL10 release by type III or type V GBS-infected DCs was MyD88-independent. No differences in DC activation were observed between NOD2^-/-^ and control DCs. These results demonstrate the involvement of various receptors and the complexity of the cytokine production pathways activated by GBS upon DC infection.

## Introduction

Group B *Streptococcus* (GBS) or *Streptococcus agalactiae* is an important cause of severe invasive bacterial infections worldwide [1]. Clinical manifestations of GBS infection include pneumonia, septicemia, and meningitis in newborns and infants. GBS diseases also occur in pregnant women, and have been recognized as an emerging cause of life-threatening invasive infections in adults, particularly the elderly and immunocompromised patients [2]. Clinical isolates of GBS are covered by a capsular polysaccharide (CPS) recognized as the most important factor for bacterial survival within the host [3]. Among ten GBS CPS types that have been characterized [1], [2], [4], type III GBS is the most common type in GBS meningitis [1]. A cohort study suggested that the high invasiveness of this serotype may be related, at least in part, to inadequate maternal and infant levels of type III CPS-specific antibodies [5]. Type V GBS has long been recognized as a leading cause of invasive disease in adults [2], [6]. To date, there are no guidelines for the prevention of adult GBS disease; vaccines in development may hold promise [6]. In a cross-sectional study analyzing older adult subjects [7], impaired type V GBS killing was associated with a low concentration of CPS-specific antibodies as well [7], [8].

Different types of leukocytes accomplish dedicated tasks in antigen presentation and killing of pathogens [9]. Dendritic cells (DCs) are recognized as the most powerful antigen-presenting cells (APCs) that initiate immune responses against pathogens and are considered an essential link between innate and adaptive immunity. In fact, DCs principal function is to alert the immune system, not to clear invading microorganisms. DCs capture and process antigens, and then undergo a maturation process characterized by the production of cytokines and up-regulation of co-stimulatory molecules. Afterwards, DCs migrate to adjacent lymphoid organs where they activate T cells [10]. The interactions between DCs and pathogens can, not only influence the pathogenesis of a disease, but also the magnitude and phenotype of the ensuing adaptive immune response. Recognition of pathogen-associated molecular patterns (PAMPs) by DCs is mediated by pattern-recognition receptors (PRRs), including the Toll-like receptor (TLR) and nucleotide-binding oligomerization domain (NOD)-like receptor (NLR) families [11]. Most TLRs are transmembrane proteins for sensing extracellular pathogens whereas NLRs sense PAMPs in the cytosolic compartment. Specially, TLR2 is reported to be specialized for the recognition of lipoproteins by generally forming a heterodimer with TLR1 or TLR6 [12], [13]. NLRs include the two-well characterized NOD1 and NOD2 [14]. NOD2 is known to sense molecules produced during the synthesis and/or degradation of bacterial peptidoglycan (PGN) and recognize muramyl dipeptide [15], a PGN constituent of both Gram-positive and Gram-negative bacteria. Interactions between TLRs and NODs with their ligands initiate an intracellular signaling cascade that induces the secretion of several pro-inflammatory cytokines and the expression of co-stimulatory cell surface molecules through the activation of transcription factors, including NF-κB [13]. Signaling occurs through association of TLRs with several adaptor molecules, such as the myeloid differentiation factor 88 (MyD88) [13]. MyD88 is utilized by all TLRs with the exception of TLR3 and drives NF-κB and mitogen-activated protein kinase (MAPK) activation to control inflammatory responses.

In contrast to other Gram-positive bacteria, TLR2 seems to play a minor role in type III GBS interactions with the host [16]–[23]. Nevertheless, among immune cells, few studies explored the role of TLRs on GBS modulation of DC functions. It has been shown that despite the importance of MyD88, TLRs 2, 4 and 9 are not involved in the production of interleukin-1 beta (IL-1β) and tumor necrosis factor alpha (TNF-α) by type III GBS-infected DCs [24]. However, TLR7 and TLR9 do recognize type III GBS nucleic acids in DC phagolysosomes after partial bacterial degradation, leading to interferon-β secretion (IFN-β) [25]. The few *in vitro* studies performed so far failed to demonstrate a clear role of NOD in types Ia and V GBS interactions with macrophages [23], [26]. We previously reported that NOD2 is not a crucial receptor to fight type III GBS infection in adult mice but the release of inflammatory cytokines in sera and by total spleen cells from GBS-infected NOD2^-/-^ mice is reduced [27]. However, no data are available concerning the role of TLR2 in GBS induction of other cytokines and chemokines, or whether different GBS serotypes interact differently with DCs. Similarly, the role of NOD2 in GBS interactions with DCs is so far unknown. In this study, we used C57BL/6 mouse bone marrow-derived DCs (bmDCs) to investigate their interactions with types III and V GBS and evaluated the capacity of these different GBS serotypes to activate DC. The potential contribution of TLR2, MyD88 adaptor protein and NOD2 in this response was assessed using bmDC generated from knock-out (KO) mice. Finally, as CPS is the most external layer at the surface of the bacteria and thus a key candidate to be involved in bacterial internalization by and interaction with DCs, the role of bacterial CPS in these processes was studied using isogenic non-encapsulated mutants.

## Materials and Methods

### Ethics Statement

All experiments involving mice were conducted in accordance with the guidelines and policies of the Canadian Council on Animal Care and the principles set forth in the Guide for the Care and Use of Laboratory Animals by the Animal Welfare Committee of the Université de Montréal. The protocols and procedures were approved by the Ethics Committee (CÉUA).

### Bacterial Strains, Plasmids and Growth Conditions

Bacterial strains and plasmids used in this study are listed in **Table 1**. Encapsulated type III GBS strain COH-1 and its isogenic non-encapsulated mutant (Δ*cpsE*) were described in previous work [25], [28]–[30]. Strain CJB111 (ATCC BAA-23) is a highly encapsulated type V GBS isolate from a neonate with septicemia [31]. An isogenic non-encapsulated type V (Δ*cpsE*) mutant was generated in this study (see below). GBS strains were grown in Todd-Hewitt Broth (THB) or agar (THA) (Becton Dickinson, Mississauga, ON, Canada) or on sheep blood agar plates at 37°C for 18 h. *Escherichia coli* strains were cultured in Luria-Bertani broth or agar (Becton Dickinson) at 37°C for 18 h. When necessary, antibiotics (Sigma-Aldrich, Oakville, ON, Canada) were added to culture media at the following concentrations: kanamycin and spectinomycin at 50 µg/ml for *E. coli*, and spectinomycin at 200 µg/ml for GBS. To perform GBS-bmDCs interaction studies, GBS strains were grown as previously described [28], [29] and diluted in complete cell culture medium prior to the experiments. The number of CFU/ml in the final suspension was determined by plating samples onto THA using an Autoplate 4000 Automated Spiral Plater (Spiral Biotech, Norwood, MA, USA). Levels of CPS production by GBS type III and type V strains were compared by chromatographic purification methods and after purity confirmation by nuclear magnetic resonance as previously described [32]. From 8 liters of GBS type III or type V culture (adjusted at O.D. values  = 0.8) the average yield of highly pure CPS was of: 59.25 mg ±17.5 (n = 4) for type III GBS and of 50.5 mg ±9.1 (n = 4) for type V GBS.

**Table 1:** Bacterial strains, plasmids and oligonucleotide primers used in this study.

Strains/Plasmids/Primers	General characteristics	Source/Reference
*Escherichia coli*		
TOP 10	F-*mrcA Δ(mrr-hsd*RMS*-mcr*BC*)φ80 lacZΔM5 Δlac*X74 *rec*A1 *ara*D139 Δ(*ara-leu*) 7697 *gal*U *gal*K *rps*L (StrR) *end*A1 *nup*G	Life Technologies Inc.
Group B *Streptococcus*		
COH-1	Wild-type, highly encapsulated strain isolated from an infant with bacteremia. Serotype III	[30]
Δ*cpsE*	Non-encapsulated strain derived from strain COH-1. Deletion of the *cpsE* gene	[28]
CJB111	Wild-type, highly encapsulated strain isolated from a neonate with septicemia. Serotype V	[31]
Δ*cpsE*	Non-encapsulated strain derived from strain CJB111. Deletion of the *cpsE* gene	In this work
Plasmids		
pCR2.1	Ap^r^, Km^r^, *oriR*(f1) MCS *oriR* (ColE1)	Life Technologies Inc.
pSET4s	Thermosensitive vector for allelic replacement. Replication functions of pG+host3 and pUC19, MCS, *lacZ*, Sp^R^	[33]
p4ΔcpsE	pSET4s carrying the construct for *cpsE* allelic replacement	In this work
Oligonucleotide primers, sequence (5′ – 3′)		
SAVE-ID1	CGGGTTTATTGTTGGTGCAGG	
SAVE-ID2	TCTTCAAGATAGCCACGACTCC	
SAVE-ID3	GCGACGCCTTAGTTTTAAGCC	
SAVE-ID4	ACGGACGATTCATCATTCCCTC	
SAVE-ID5	TGGTCGTTCCTTCAGGGAAAG	
SAVE-ID6	GCTCCTGTCCCGAGTAAAACTCCACAACTGTTTGAATCATCGC	
SAVE-ID7	GCGATGATTCAAACAGTTGTGGAGTTTTACTCGGGACAGGAGC	
SAVE-ID8	AATGGTACTGCTACAGCGGC	

### Construction of Type V GBS Non-encapsulated Mutant

GBS genomic DNA was extracted by InstaGene Matrix solution (BioRad Laboratories, Hercules, CA, USA). Minipreparations of recombinant plasmids and transformation of *E. coli* were performed by standard procedures. Restriction enzymes and DNA-modifying enzymes were purchased from TaKaRa Bio (Otsu, Shiga, Japan) and used according to the manufacturers' recommendation. PCR reactions were carried out with iProof proofreading DNA polymerase (BioRad Laboratories, Mississauga, ON, Canada). Oligonucleotide primers were from IDT technology (Coralville, IA, USA). Amplification products were purified with QIAgen PCR purification kit (QIAGEN, Valencia, CA, USA) and sequenced with an ABI 310 automated DNA sequencer, using the ABI PRISM dye terminator cycle sequencing kit (Life Technologies Inc., Burlington, ON, Canada).

**Figure 1:**
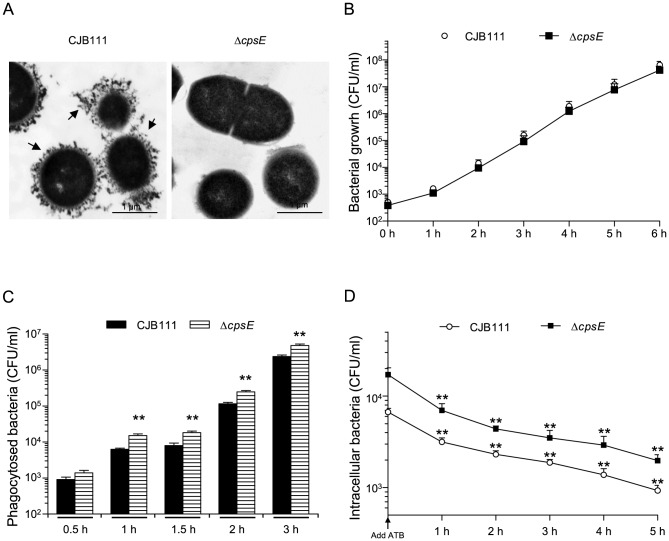
Phagocytosis by and intracellular survival within DCs of type V GBS: role of bacterial capsular polysaccharide. (A) Transmission electron micrographs of GBS strains labeled with polycationic ferritin show GBS wild-type strain CJB111 with a thick capsule (indicated by arrows) whereas no capsular material is observed in Δ*cpsE* mutant strain. (B) Growth curves of wild-type GBS strain CJB111 and Δ*cpsE* mutant strain. (C) Wild-type type V GBS strain CJB111 or the Δ*cpsE* non-encapsulated mutant (10^6^ CFU/ml, initial MOI:1) were incubated with C57BL/6J-derived bmDCs for different time periods. Internalized bacteria were enumerated by quantitative plating after 1 h of antibiotic treatment to kill extracellular bacteria. ** *P*<0.01, indicates statistically significant differences between the wild-type strain CJB111 and the non-encapsulated mutant, n = 5. (D) For intracellular survival assays, bmDCs were infected with GBS strains (MOI:1) and phagocytosis was left to proceed for 60 min. Antibiotics (ATB) were then added for 1 h (defined as time 0). This initial antibiotic-treatment was extended up to 5 h and cells lysed to quantify intracellular bacteria by viable plate counting. ** *P*<0.01, indicates incubation times for which significantly differences in the numbers of recovered intracellular bacteria were observed compared to time 0, n = 8. All results are expressed as CFU recovered bacteria per ml (means ± SEM). It should be noted that initial MOI was the same for all conditions and bacterial growth rate in the culture medium was identical for both strains.

The DNA sequence of type V GBS strain CJB111 capsular (*cps*) locus was retrieved from GenBank (Accession numbers AAJQ01000091.1 and AAJQ01000044.1) and used as sequence template for primers design. Precise deletion of the *cpsE* (1304 bp) gene was constructed by using splicing-by-overlap-extension PCR and the primers listed in **Table 1**. The PCR-generated Δ*cpsE* deletion allele was cloned into plasmid pCR2.1 (Invitrogen), extracted with EcoRI and recloned into the thermosensitive shuttle plasmid pSET4s [33] digested with the same enzyme, giving rise to the knockout vector p4ΔcpsE. Electroporation of GBS with the recombinant plasmid and procedures for isolation of mutants were those described previously [28]. Allelic replacement was confirmed by PCR and sequencing analysis. The non-encapsulated (Δ*cpsE*) phenotype of the mutant was confirmed by absence of reaction in the coagglutination test using rabbit antisera against type V GBS capsular material (Denka Seiken, Campbell, CA, USA), and by transmission electron microscopy using polycationic ferritin labeling as previously described (**Fig. 1A**) [28]. Growth rates were not significantly affected in the mutant strain compared to wild-type (WT) GBS (**Fig. 1B**).

### Generation of Bone Marrow-derived Dendritic Cells (bmDCs)

BmDCs were generated from six to eight week-old female mice originated from Jackson Laboratory (Bar Harbor, ME, USA), including control (CTRL) C57BL/6J, MyD88^−/−^ (B6.129P2-*Myd88^tm1Defr^*/J), TLR2^−/−^ (B6.129-*Tlr2* ^tmlKir^/J) and NOD2^−/−^(B6.129S1-*Nod2^tm1Flv^*/J). BmDCs were produced according to a technique described elsewhere [28], [29]. Briefly, after red blood cell lysis, total bone marrow cells (2.5×10^5^ cells/ml) were cultured in complete medium consisting of RPMI 1640 supplemented with 5% heat-inactivated fetal bovine serum, 10 mM HEPES, 20 µg/ml gentamicin, 100 U/ml penicillin-streptomycin, 2 mM L-glutamine and 50 µM 2-mercaptoethanol. All reagents were from Gibco (Life Technologies Inc.). Complete medium was complemented with 20% GM-CSF from a mouse GM-CSF transfected cell line (Ag8653) as a source of GM-CSF [34]. Cells were cultured for 7 days at 37°C with 5% CO_2_. On day 7, clusters were harvested and subcultured overnight to remove adherent cells. Non-adherent cells were collected on day 8 and used as immature bmDCs for the studies. Cell purity was routinely ≧88% CD11c^+high^ cells as determined by FACS analysis and as previously reported [28].

### Bacterial Internalization Assays

BmDCs at a concentration of 10^6^ cells/ml were infected with 10^6^ CFU/ml of WT type V GBS or its non-encapsulated mutant strain (initial MOI:1), and incubated for 0.5 to 3 h at 37°C with 5% CO_2_ (without addition of exogenous complement). MOI and assay conditions were chosen based on previous studies on the kinetics of type III GBS phagocytosis by DCs [28]. After incubation, 100 µg/ml of gentamicin and 5 µg/ml of penicillin G (Sigma-Aldrich) were added to kill extracellular bacteria. After 1 h-antibiotic treatment, cells were washed 3 times with PBS, lysed with sterile water and viable intracellular streptococci enumerated by quantitative plating of serial dilutions of the lysates on THA.

For intracellular survival studies, internalization assays were performed as described above, except that after a 60 min initial bacterial-cell contact, gentamicin-penicillin were added and the treatment was lengthened for different times up to 5 h. The results were expressed as CFU/ml of recovered intracellular viable bacteria.

### *In Vitro* bmDC Stimulation Assay

BmDCs were resuspended at 10^6^ cells/ml in complete medium and stimulated with different GBS strains (10^6^ CFU/ml; initial MOI:1). Conditions used were based on those already published [28]. After 2 h of bmDC-GBS infection, the bacteriostatic agent chloramphenicol (CM, 12 µg/ml, Sigma-Aldrich) was added to the culture to prevent cell toxicity as previously reported [28]. After 16 h of incubation, supernatants were collected for cytokine quantification by ELISA. Non-stimulated cells served as negative control. The specific TLR2-ligand PAM(3)CSK(4) (at 0.5 µg/ml [Invivogen, San Diego, CA, USA]), the TLR4-ligand lipopolysaccharide (ultra-pure LPS, at 1 µg/ml [Apotech Corporation, Epalinges, Switzerland]), and the specific NOD2-ligand muramyl dipeptide (MDP, at 10 µg/ml [Invivogen]) were used as controls (**Fig. S1**). Lactate dehydrogenase (LDH) release measurement assay was used to confirm absence of cytotoxicity in bacterial-bmDC cultures (Promega CytoTox96, Promega Corporation, Madison, WI, USA) [28].

### Cytokine Quantification by ELISA

Levels of IL-6, IL-10, IL-12p70, IL-23p19, TNF-α, CCL2 (MCP-1), CCL3 (MIP-1α) CXCL1 (KC) and CXCL10 (IP-10) in cell culture supernatants were measured by sandwich ELISA using pair-matched antibodies from R&D Systems (Minneapolis, MN, USA) or eBioscience (San Diego, CA, USA), according to the manufacturer's recommendations. Twofold dilutions of recombinant mouse cytokines were used to generate standard curves. Sample dilutions giving OD readings in the linear portion of the appropriate standard curve were used to quantify the levels of each cytokine.

### Statistical Analysis

All data are expressed as mean ± SEM. Data were analyzed for significance using ANOVA combined with Bonferroni t-test for correction. A *P* value <0.05 was used as a threshold for significance. All experiments were repeated at least three times.

## Results and Discussion

### DCs Efficiently Internalize Both the Encapsulated and the Non-encapsulated type V GBS Strains

We demonstrated previously that encapsulated type III GBS is easily internalized by DCs, but efficiently survives inside these cells for at least 6 h [28]. The CPS only slightly and transitorily affects type III GBS internalization at short incubation times (1 h). On the other hand, the type III CPS plays a role in modulating bacterial survival within DCs [28]. To date, despite interactions with human epithelial and endothelial cells [35]–[39], mouse macrophages or human neutrophils [23], [40]–[42], no data are available on the phagocytosis capacity of bmDCs face to type V GBS or on the importance of the type V CPS in modulating this activity. As shown in **Fig. 1C**, the encapsulated CJB111 strain was rapidly internalized by bmDCs. Phagocytosis levels increased over time reaching levels as high as 10^7^ CFU of total recovered intracellular bacteria at 3 h. Longer incubation times could not be tested, as GBS type V was toxic to bmDCs (by LDH assay, data not shown). After 1 h of incubation, the Δ*cpsE* mutant was significantly more internalized than the WT strain and this difference was observed at least until 3 h of incubation (*P<*0.01). Survival assay (**Fig. 1D**) showed a slow decrease in WT GBS intracellular survival, which was statistically significant after 1 h of GBS post-internalization (*P<*0.01), with ∼2×10^3^ CFU of remaining live intracellular bacteria at the end of the survival test. Although the non-encapsulated mutant strain was more internalized than the WT strain, the intracellular survival rates remained similar to those of the parental strain.

Compared to type III GBS, type V CPS slightly prevented the internalization of GBS overtime, but does not seem to protect bacteria from intracellular killing. GBS is serologically classified into ten distinct serotypes (Ia, Ib, II-IX) based on antigenic differences in the CPS [3]. The structures of the ten CPS types are similar in their constituent monosaccharide compositions and certain structural motifs, yet they differ sufficiently to be antigenically distinct. The polysaccharide repeat unit structures of types III and V are not closely related and may explain the observed differences [3]. Variations in the thickness of CPS expressed by the strains used in this study cannot be completely ruled out, albeit CPS yields seem to be similar between the two strains (as aforementioned). Finally, intrinsic differences related to these two particular strains and independent of the serotype might also influence bacterial phagocytosis.

### Internalization of GBS is Independent of TLR and NOD2 Receptor Signaling

**Figure 2:**
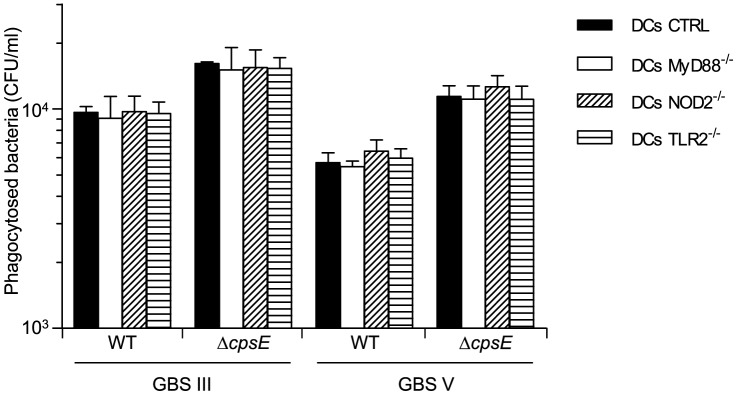
Effect of MyD88, TLR2 or NOD2 deficiency on the capacity of DCs to internalize GBS. Control (CTRL), MyD88^-/-^, TLR2^-/-^ or NOD2^-/-^ bmDCs were incubated for 1 h with GBS wild-type (WT) strains or their respective non-encapsulated (Δ*cpsE*) mutants (10^6^ CFU/ml, initial MOI:1). Internalized bacteria were enumerated by quantitative plating after 1 h of antibiotic treatment to kill extracellular bacteria (n = 5).

Albeit controversial, it has previously been reported that TLRs may be involved as receptors for bacterial phagocytosis [43], [44]. The absence of TLR2 delayed *Streptococcus pneumoniae* phagocytosis and killing by neutrophils [45]. In contrast, TLRs were shown not to play a significant role in phagocytosis of type III GBS by peritoneal macrophages [20] and *Streptococcus suis* by DCs [46]. On the other hand, it has been observed that NOD1- or NOD2-deficient mice have decreased phagocytic abilities [47], [48], in particular in neutrophils. Yet, the influence of NOD receptors on phagocytosis does not seem to be universal [49], [50]. So, in this study, GBS internalization by DCs was evaluated by the antibiotic-protection phagocytosis assay using bmDCs from MyD88^-/-^, TLR2^-/-^ and NOD2^-/-^ mice. As displayed in **Fig. 2**, no significant differences were observed in bmDC capacity to internalize WT GBS strains in the absence of MyD88, TLR2 or NOD2. The difference in phagocytosis levels between WT strains and their respective non-encapsulated mutants remained the same in deficient-bmDCs compared to CTRL cells. Similarly, no differences were observed in the intracellular fate of GBS strains between CTRL and KO cells (data not shown).

### DC Activation by GBS Infection and the Importance of MyD88 Adaptor Protein is not Serotype Restricted

**Figure 3:**
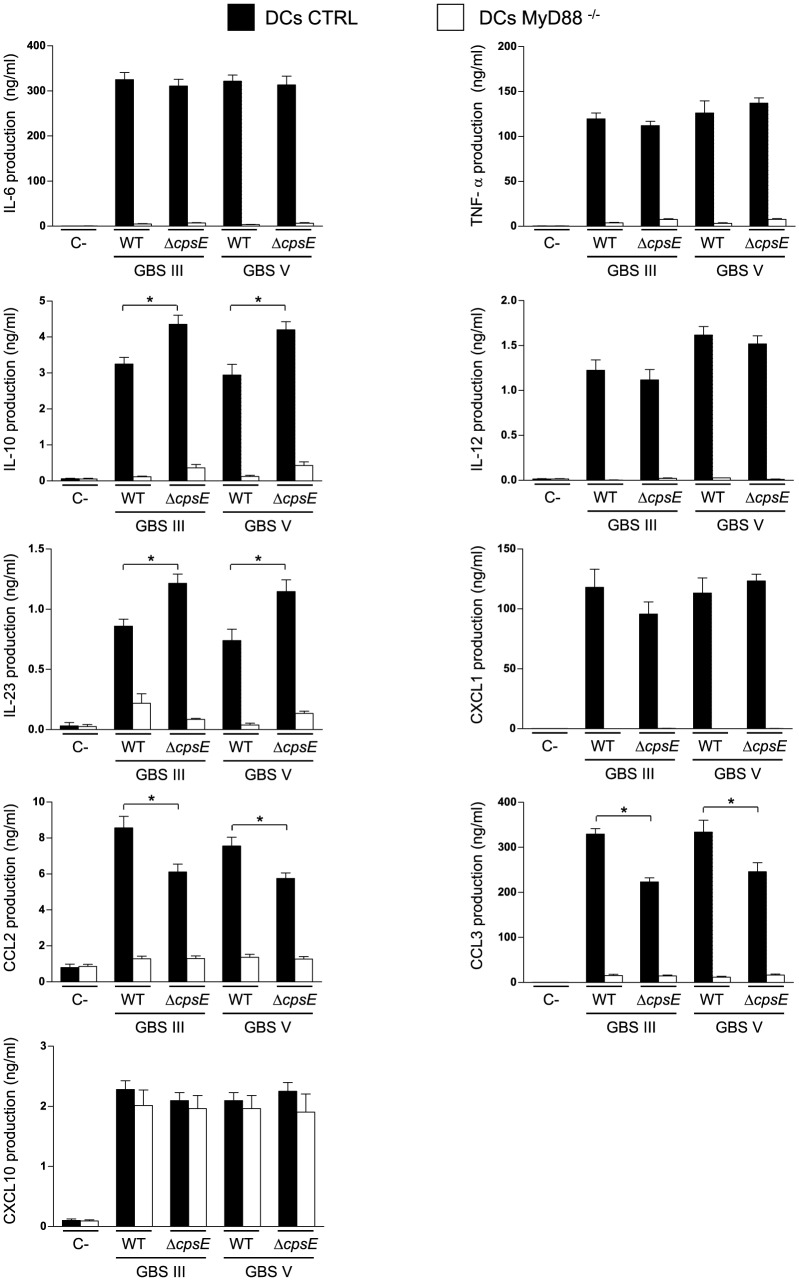
Impact of MyD88 on cytokine release by DCs in response to GBS. Control (CTRL) or MyD88^-/-^ bmDCs were stimulated with GBS wild-type (WT) strains or their respective non-encapsulated (Δ*cpsE*) mutants (10^6^ CFU/ml, initial MOI:1). After 2 h of bmDC-GBS infection, a bacteriostatic agent (chloramphenicol, 12 µg/ml) was added to the culture to prevent cell toxicity. Non-stimulated cells served as negative control (C-) for basal expression levels. Supernatants were harvested at 16 h of incubation and cytokine production quantified by ELISA. Data are expressed as mean ± SEM (in ng/ml) from eight independent experiments. * *P*<0.05, indicate statistically significant differences between WT strains and their respective non-encapsulated mutants.

As MyD88 adaptor protein mediates numerous biologically important signal transduction pathways in innate immunity [51], its contribution to DC cytokine production following stimulation with GBS was investigated. BmDCs from CTRL or MyD88^-/-^ mice were incubated with different GBS strains for 16 h. Optimal assay conditions were chosen based on previous results [28] and preliminary studies on the kinetics of cytokine release by bmDCs in response to GBS (data not shown). As we previously reported [28], [29], type III GBS-activated DCs produced significant levels of the pro-inflammatory cytokines IL-6 and TNF-α, the Th1-driving cytokine IL-12p70, the regulatory cytokine IL-10, and the chemokines CCL2, CXCL1 and CXCL10 (**Fig. 3**). The encapsulated type V GBS strain released similar levels of these cytokines than the encapsulated type III GBS strain. BmDCs were also able to produce significant amounts of the Th17-driving cytokine IL-23 and high levels of another member of the CC chemokine family, CCL3, after exposure to either types III or V GBS. In general, the non-encapsulated mutants produced similar levels of cytokines and chemokines compared to respective WT strains (**Fig. 3**). Yet, the absence of CPS resulted in higher levels of IL-10 and IL-23 production by bmDCs stimulated with either serotype. We previously reported that the absence of CPS affects CCL2 production by type III GBS-infected DCs [29]. The same phenotype was observed here for type V GBS. Furthermore, CCL3 production was also significantly attenuated in the non-encapsulated mutant strains for both GBS serotypes (**Fig. 3**). Thus, types III and V GBS induce similar patterns of cytokine production by DCs and this response is similarly affected by the presence of CPS in both serotypes.

With the exception of CXCL10, the production of cytokines and chemokines by bmDCs was either completely abrogated or markedly impaired in MyD88^−/−^ bmDCs for all strains tested, independently of the CPS serotype (**Fig. 3**, *P<*0.001). It was demonstrated that LPS activation of MyD88^-/-^ mouse macrophages results in impaired gene expression of various chemokines but not of CXCL10 compare to CTRL cells [52]. In contrast, MyD88 was partially involved in CXCL10 release by DCs exposed to the encapsulated pathogen *S. suis* [46].

### TLR2 Has a Partial Role in DC Activation in Response to GBS Infection

**Figure 4:**
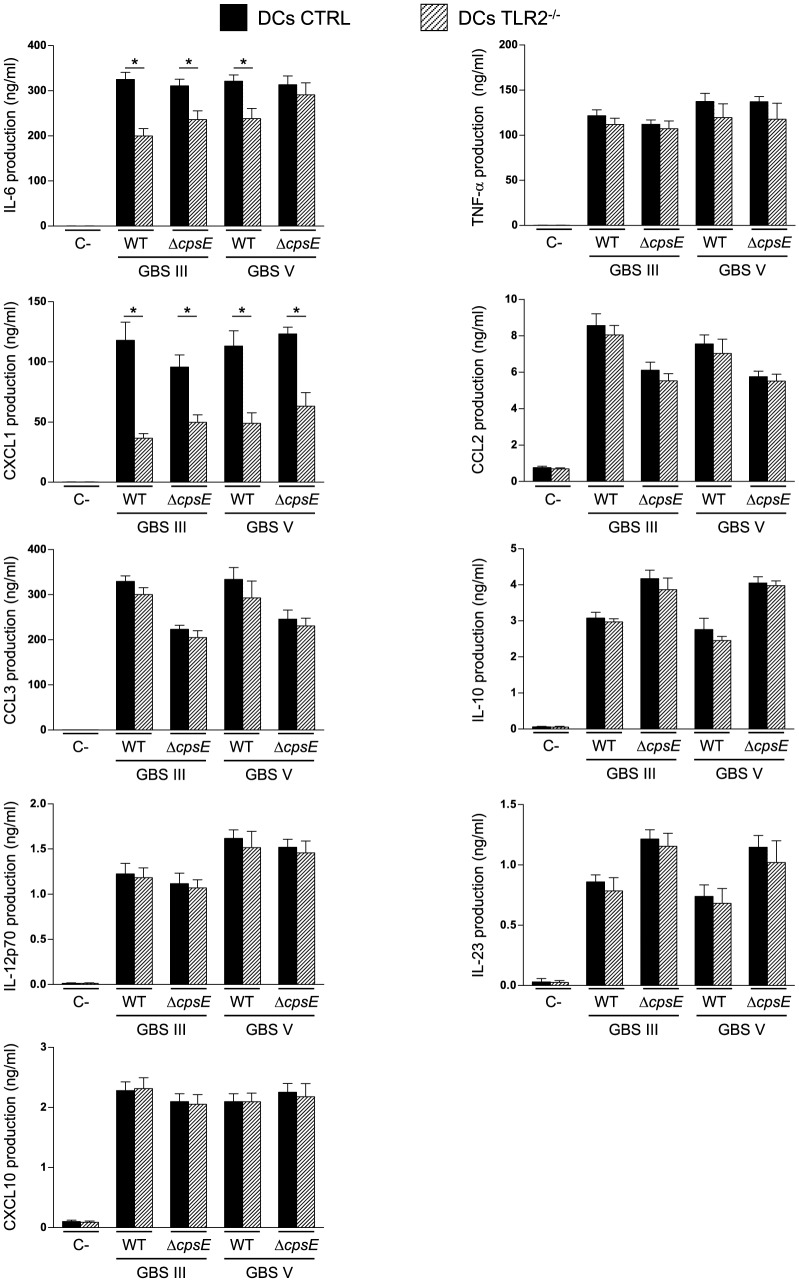
Effect of TLR2 on cytokine release by DCs in response to GBS. Control (CTRL) or TLR2^-/-^ bmDCs were stimulated with GBS wild-type (WT) strains or their respective non-encapsulated (Δ*cpsE*) mutants (10^6^ CFU/ml, initial MOI:1). After 2 h of bmDC-GBS infection, a bacteriostatic agent (chloramphenicol, 12 µg/ml) was added to the culture to prevent cell toxicity. Non-stimulated cells served as negative control (C-) for basal expression levels. Supernatants were harvested at 16 h of incubation and cytokine production quantified by ELISA. Data are expressed as mean ± SEM (in ng/ml) from eight independent experiments. * *P*<0.05, indicate statistically significant differences between CTRL and TLR2^-/-^ bmDCs.

The involvement of TLR2 in DC cytokine production following stimulation with GBS was also investigated using TLR2^-/-^ bmDCs. We have previously demonstrated that the production of various cytokines by type III GBS-stimulated DCs is either partially-dependent or highly-dependent on bacterial internalization [28], [29]. As TLR2 was not involved in GBS internalization, we firstly focused on those cytokines that seem to be mainly triggered upon GBS contact with a cell surface receptor (IL-6, TNF-α, CXCL1, CCL2 and CCL3 [28], [29]). As shown in **Fig. 4**, only the release of IL-6 and CXCL1 was partially reduced in TLR2^-/-^ bmDCs infected by encapsulated GBS type III or type V strains (*P*<0.05). The non-encapsulated mutant of type III GBS showed a similar dependency on TLR2 than the WT strain. In contrast, IL-6 release by bmDCs stimulated with the non-encapsulated mutant of type V GBS was not affected by the absence of TLR2, yet this response was markedly MyD88-dependent (**Fig. 3**). It is thus likely that high levels of surface exposition of cell wall components (normally hidden by the type V CPS) are able to activate cells through multiple TLRs.

The production of the cytokines IL-10, IL-12p70, IL-23 and CXCL10, known to be dependent on GBS internalization ([28] and unpublished data), was also evaluated and, as expected, no differences were observed in the capacity of TLR2^-/-^ bmDCs to produce these cytokines compared to CTRL cells after either type III or type V GBS infection (**Fig. 4**).

Previous *in vitro* studies proposed that TLR2 is involved at a certain level in GBS-induced cell activation and cytokine production. Similarly to our data, the expression of a major fraction of genes in macrophages induced by whole heat killed-type III GBS does not significantly depend on TLR2, such as *tnf*, *Ccl2*, *Ccl3*, *Il-12*, and *Cxcl10*. On the other hand, and in agreement with features observed with DCs, induction of only few important molecules involved in host innate immunity, such as IL-6 and IL-1β, was impaired in the absence of TLR2 signaling in macrophages [18]. Another study reported that activation of peritoneal macrophages by whole type III GBS is independent of TLR2 and TLR6, whereas a response to the secreted heat-labile soluble factor released by type III GBS was dependent on TLR2 [17]. Furthermore, lipoteichoic acid from type III GBS is recognized as a TLR2/TLR6 ligand, but does not contribute significantly to GBS cell wall mediated macrophage activation [19]. Albeit induction of IκB kinase activation (the kinase regulating NF-κB activation) but not that of JNK and p38 MAPKs in type V GBS-infected macrophages is blocked in the absence of MyD88, it is only partially inhibited in the absence of TLR2 [23]. Finally, a study using human TLR2-transfected fibroblast cell line failed to demonstrate a role of TLR2 in cell interactions with heat killed-type III GBS compared to *Listeria monocytogenes* [21]. In agreement with our results, Costa *et al.* have shown that TLR2 is not responsible for the release of TNF-α by type III GBS-infected bmDCs [24]. In contrast, it has been demonstrated *in vivo* for both type III and type V GBS that TLR2/MyD88 mediates TNF-α and IL-6 production and contributes to bacterial clearance in a low-dose sepsis model, whereas it is detrimental in a high-dose model of septic shock due to an enhanced inflammatory response [22]. In contrast, another study reported that TLR2^-/-^ mice infected with type IV GBS show earlier and higher mortality rates and increased incidence and severity of disease than control mice at all the infecting doses employed [53]. Recently, Andrade *et al.* demonstrated that TLR2-induced IL-10 production is a key event in neonatal susceptibility to type III GBS sepsis [54]. Thus, the role of TLR2 in GBS immuno-pathogenesis remains controversial and seems to depend on the infection model, the bacterial dose and/or the GBS serotype used. Yet, overall, TLR2 does not seem to play a major role in GBS activation of immune cells.

### NOD2 Does Not Seem to Play a Role in DC Activation in Response to GBS Stimulation *In Vitro*

**Figure 5:**
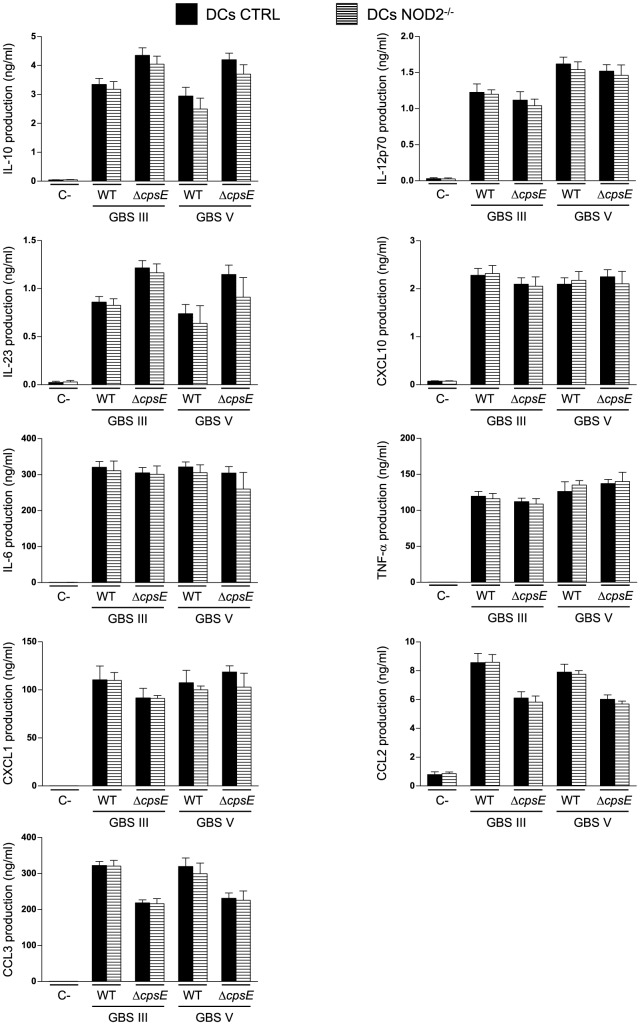
Effect of NOD2 on cytokine release by DCs in response to GBS. Control (CTRL) or NOD2^-/-^ bmDCs were stimulated with GBS wild-type (WT) strains or their respective non-encapsulated (Δ*cpsE*) mutants (10^6^ CFU/ml, initial MOI:1). After 2 h of bmDC-GBS infection, a bacteriostatic agent (chloramphenicol, 12 µg/ml) was added to the culture to prevent cell toxicity. Non-stimulated cells served as negative control (C-) for basal expression levels. Supernatants were harvested at 16 h of incubation and cytokine production quantified by ELISA. Data are expressed as mean ± SEM (in ng/ml) from eight independent experiments.

We have previously reported that for type III GBS, the release of IL-10, IL-12p70, CXCL10 [28] and IL-23 is dependent on bacteria internalization by bmDCs (data not shown). Thus, we evaluated the role of NOD2, an important intracellular receptor, in the production of these cytokines by bmDCs upon infection with either type III or type V GBS. **Fig. 5** shows that NOD2^-/-^ bmDCs produced the same amount of IL-10, IL-12p70, CXCL10 and IL-23 as CTRL bmDCs did when infected by either encapsulated type III or type V GBS, or by their respective non-encapsulated mutants. No differences were observed between CTRL bmDCs and NOD2^-/-^ bmDCs in the release of other evaluated cytokines (**Fig. 5**; *P*>0.05).

*In vitro* studies performed with receptor-interacting protein 2 (RIP2) knockout macrophages, which lack NOD1 and NOD2 signaling, showed that both heat-killed and live type Ia GBS activate a potent IFN-β response similar to that of CTRL cells [26]. Other study reported that NOD2 is dispensable for IκB kinase and MAPK activation in type V GBS-infected macrophages [23]. In contrast, e*x vivo* analysis of total spleen cells from type III GBS-infected mice showed that the absence of NOD2 results in reduced production of inflammatory cytokines [27]. Nevertheless, results presented here suggest that DCs do not contribute to this NOD2-dependent inflammatory response and that, instead, other immune cells would be involved. For example, it has been shown that NOD1 and NOD2 are necessary for optimal IFN-γ production by iNKT cells, as well as NK cells [55].

It has been shown that *S. pneumoniae* induces production of inflammatory cytokines in primary murine microglia, astrocytes and bone marrow-derived macrophages in a NOD2-dependent manner [56], [57]. In the case of *S. suis*, Lecours *et al.* observed that the production of IL-23 and CXCL1 by infected-DCs is partially dependent on NOD2 [46]. Our results seem to indicate that other intracellular receptors might be implicated in induction of cytokine release upon GBS internalization. Costa *et al.* reported that activation of the inflammasome, an inflammatory signaling complex, by type III GBS mediates *in vitro* production of IL-1β and IL-18, but not of TNF-α by DCs. Activation of the NLRP3 inflammasome requires GBS expression of β-hemolysin, an important virulence factor [24]. In addition, TLR7 and TLR9 also recognize type III GBS nucleic acids in DC phagolysosomes after partial bacterial degradation, leading to IFN-β secretion [25]. TLR9 sensing of type Ia GBS DNA was reported to be involved in the upregulation of TNF-α, IL-6 and IL-12 by mouse macrophages [58]. In our study, the release of IL-10, IL-12p70 and IL-23 was shown to be MyD88-dependent. We speculate that TLR7 and TLR9 receptors are also implicated in the production of these cytokines by type III and type V GBS once internalized by DCs.

## Conclusions

Progress has been achieved in recent years regarding our understanding of the complex interactions between GBS and DCs, especially in the context of type III GBS infection. However, due to limited information, it was unclear whether these interactions are similar or different among the diverse GBS serotypes. Here we demonstrated that type V GBS CPS partially impairs bacterial internalization, but does not improve bacterial intracellular survival, a pattern that slightly differs from that of type III GBS CPS. Interestingly, our results show that for both serotypes the TLR/MyD88 and NOD2 pathways do not modulate bacterial internalization. The TLR/MyD88 signaling cascade plays a major role in cytokine production by type III and type V GBS-infected DCs. Yet, TLR2 only partially contributes to DC activation by GBS of both serotypes. Upon internalization by DCs, both GBS types induce production of IL-10, IL-12p70 and IL-23 by a NOD2-independent but MyD88-dependent pathway, probably via an intracellular TLR. Notably, the production of the chemokine CXCL10 was shown to be MyD88/NOD2-independent. Induction of type I interferons and their regulatory pathways are probably involved in CXCL10 production. This pathway might involve TRIF activation instead of MyD88, but this remains a working hypothesis [59]. Yet, a possible MyD88/TRIF-independent pathway for type I interferon production, as reported with GBS-infected macrophages cannot be ruled out [26]. Altogether, our data support the hypothesis that GBS use complex TLR/MyD88-dependent in addition to TLR/NOD2-independent pathways to modulate host immune responses mediated by murine DCs.

In our model, the CPS only partially influences type III and type V GBS interactions with DCs. In fact CPS, as an antigen, only contributes to CCL2 and CCL3 production by DCs stimulated by either GBS serotype. In agreement with these results, these chemokines are the only ones produced by DCs in contact with highly purified type III or type V GBS CPS [32]. In this study, CPS-mediated production of CCL3 was shown to be partially via TLR2 and MyD88-dependent pathways whereas CPS-induced CCL2 production involves TLR-independent mechanisms. Overall, no major differences were observed between type III and type V GBS in their interactions with DCs. However, further studies using multiple strains for each serotype are required to dissect the role of serotype in DC responses to GBS.

Albeit DCs and macrophages are immunologically distinct in their specific functions, surface receptors involved in GBS recognition by DCs seems to be similar to those reported in GBS interactions with macrophages. MyD88 signaling pathways are largely involved in GBS activation of both cell types, but TLR2 seems to play a minor role [17], [20], [60], [61]. In contrast to surface receptors, intracellular sensing of GBS-derived molecules, including RNA and DNA, seems to occur in a cell-lineage specific manner, as reported by previous works [25], [26], [58], [61]. All published studies addressing the interactions of GBS with DCs have been performed with murine-origin cells; it remains thus unclear how closely the activation of human DCs by GBS resembles the activation patterns observed with murine cells.

## Supporting Information

Figure S1 **Cytokine response of knock-out cells using specific TLR-ligands.** Control (CTRL), MyD88-/-, TLR2-/- or NOD2-/- bone marrow-derived dendritic cells (DCs) were stimulated with ultra-pure lipopolysaccharide (LPS, 1 µg/ml), PAM(3)CSK(4) (0.5 µg/ml) or muramyl dipeptide (MDP, at 10 µg/ml). Non-stimulated cells served as negative control (C-) for basal expression levels. Supernatants were harvested at 16 h of incubation and cytokine production quantified by ELISA. Data are expressed as mean ± SEM (in ng/ml) from four independent experiments. ** *P*<0.01, indicates statistically significant differences between CTRL and knock-out cells at the same condition.
(EPS)
